# 

**Published:** 1887-06

**Authors:** 


### INDEX TO VOLUME LIV.

A.

PAGE

Abscess in the Brain, Resulting from Disease of the Ear...... 511
A Compend of Electricity and its Medical and Surgical Uses, Mason.................... 544
A Compend of Pharmacy, Stewart.................... 331
Adenoma, Ovarian, with Uterus Suppurating.............. 173
Adam, T. B., A Case of Reynaud's Disease............. 1
A Laboratory Guide in Urinalysis and Toxicology, Witthaus 331
American Journal of Biology.. 211
American Medical Association, Annual Meeting of the..... 323
Antiphyrin and Thallin.... 10–195
Aneurism, Thoracic........... 111
Annual Report of the Museum of Hygiene of the United States Navy............... 305
Annual Meeting of the Italian Surgical Society............ 456
A Practical Treatment on Obstetrics.................... 633
A Treatise on Obstetrics...... 632
A Treatise on Diseases of the Skin, with Special Reference to their Diagnosis and Treatment...................... 633
A Text-Book of Pathological Anatomy and Pathogenesis. 633
A Text-Book of Medicine for Students and Practitioners, Strümpell................. 540
A Text-Book on Surgery, General, Operative and Mechanical, Wyeth................ 541
Artificial Illumination........ 240
Arlt, Professor, Obituary...... 533
Asthma, Etiology and Cure of. 279

PAGE

Autopsies, Two Recent, at Cook County Hospital............ 416

B.

Barr, Thomas, Abscess of the Brain Resulting from Disease of the Ear................ 511
Bartlett, Dr. John, The Warming Crib................. 449
Belfield, W. T., Prostatic Myoma Removed by Supra-Pubic Prostatomy............... 402
Mucous Casts from a Case of Membranous Enteritis 403
Foreign Body from the Bladder............... 403
Fragment of Occipital Bone................. 403
Digital Exploration of the Kidney............... 476
Belfield, W. T., Intestine covered with Miliary Tubercles from Case of Miliary Tubercular Meningitis.......... 404
Bettman, B., Recurrent Hæmorrhage into the Anterior Chamber...................... 169
Connection between Ocular and Nasal Diseases.. 290
Biology, American Journal of. 211
Bladder, a Case of Foreign Body in the............... 403
Book Reviews and Notices.... 90
...............209, 236, 438, 540
Books Received............332, 446
Brand, Ernst, Concerning the Present Standing of the Water Treatment of Typhoid Fever.................... 489
Brain, Abscess of, Resulting from Disease of the Ear... 511
Bronchitis, Chronic Pseudo-Membranous........30, 139, 201

### C.

PAGE

Calcareous Concretions in the External Ear ---- 464

Caldwell, W. S., Surgery in the Continent of Europe ---- 148

Medicine in Paris and Vienna.

Cancer, a Study of Three Hundred and Ninety-seven Cases, Willard Parker ---- 541

Case of Perinephritic Abscess, 370

Cataract, Comparison of the Methods of Removal of, with and without Iridectomy ---- 525

Chancre of the Eyelid ---- 467

Chest Movements of Respiration in the Indian Female ---- 521

Chicago Gynæcological Society, Meeting, Nov. 19, 1886 ---- 69

Dec. 17, 1886 ---- 171

Jan. 21, 1887 ---- 259

Feb. 18, 1887 ---- 373

March 18, 1887 ---- 479

Chicago Society of Ophthalmology and Otology, Meeting, Dec. 14, 1886 ---- 161

Feb. 8, 1887 ---- 618

Feb. 12, 1881 ---- 620

Chicago Medical Society, Meeting, Nov. 15, 1886 ---- 61

Dec. 6, 1886 ---- 195

Dec. 20, 1886 ---- 201

Jan. 3, 1887 ---- 279

Jan. 17, 1887 ---- 288

Feb. 21, 1887 ---- 396

March 7, 1887 ---- 405

M'rch 21, 1887 ---- 467

April 18, 1887 ---- 604

Climatic Treatment of Disease, ---- 299, 396

Cocaine-Dosage and Cocaine-Addiction ---- 335

Cocaine, Untoward Effects of ---- 522

Commencement Exercises of the Medical Colleges of Chicago ---- 424

Conjunctivitis, a Form of Gonorrhœal, not Dependent upon Inoculation ---- 524

Congress, Ninth International, Medical. See Medical Congress, etc ----

Curtis, Lester, The Absence of the Patella Tenden Reflex ---- 64

Cyst, Parovarian and Appendages, etc. ---- 172

PAGE

Cyst, Ovarian in Broad Ligament Containing Degenerated Ovary ---- 172

Cystonia, Proliferating Ovarian.

### D.

Dalby, Sir William B., on the Management of Perforations of the Membrana Tynpani ---- 527

Davis, Dr. N. S., Celebration of the Semi-Centennial of His Entry into the Medical Profession ---- 146

Disinfecting Solutions, Recommended by the State Board of Health of Pennsylvania ---- 434

Diseases of the Joints, Marsh ---- 540

Disease of the Sound-Conducting and Sound-Perceiving Apparatus ; The Differential Diagnosis of ---- 576

Diet and Hygiene in the Treatment of Diabetes ---- 558

Dental Review, The ---- 211

Drug Eruptions : A Clinical Study of the Effect of Drugs upon the Skin ---- 630

Dudley, E. C., Gynæcological Antiseptics ---- 228

### E.

Enteritis, a Case of Membranous ---- 403

Elementary Microscopical Technology: A Manual for Students ---- 631

Electrolysis in the Treatment of Fibroid Tumors of the Uterus ---- 203

Eighth Annual Report of the Illinois State Board of Health 209

Earl, P., The Curability of Insanity ---- 90

Epilepsy : Electricity in the Treatment of ---- 627

Essentials of the Physical Diagnosis of Thoracic Diseases 442

### F.

Fevers : Their History, Etiology, Diagnosis, Prognosis and Treatment ---- 632

Fenger, Christian, The Operative Treatment of Retropertoneal Cysts in connection with Mikulicz' Method of Drainage ---- 481

PAGE

Fibro-Sarcoma of Uterus, Lungs and other organs 174
Foreign Body in the Anterior Chamber 289
Fracture of Occipital Bone, a Case of 403
Franklin, W., Sympathetic Opthalmia 291

G.

Gihon, A. L., Chronic Diffuse Nephritis 15
Glancoma, A Clinical Study of 288
Genococcus of Neiser, The 573
Gradle, H., Diseases of the Vault of the Pharynx 18
Guy's Hospital Reports 443

H.

Hand-Book of the Diseases of the Nervous System 441
Hand-Book of Practical Medicine, Eichhorst 327
Hand-Book of Practical Medicine 440
Gynæcological Antiseptics 228
Hearing and Examinations for the Public Service 556
Heart with Atheroma, Exhibition of 285
Hæmorrhage, Ante-partum, at Term, a Case of 286
Herpes Omo-Brachialis, Case of 237
Hoadley, A. E., Deep-Tubing of the Larynx as a Substitute for Intubation 407
Holmes, E. L., Foreign Body in the Anterior Chamber 289
A Case of Intra-Ocular Tumor 290
A Case of Chancre of the Eyelid 467
Honors to a Worthy Practitioner 627
Hospital Appointments in Chicago for the Current Year 423
Hot Springs Hospital 432
House Plants as Sanitary Agents, Anders 332
Hydrophobia Vera, with report of a Case 544
Hystero—Neurasthenia 405

I.

PAGE

Ichthyol in Treatment of Skin Diseases 66
Ilium of Child, Showing Characteristic Lesions of Typhoid Fever 176
Illinois State Medical Society, Annual Meeting of 577
Index Catalogue of the Library of the Surgeon-General's Office 445
In How Many Ways Do we Hear? 571
Insanity, The Curability of, P. Earl 90
Intubation Instruments Modified 300
Intussusception in Infants 469

J.

Jackson, A. Reeves, A Case of the Removal of the Ovaries and Tubes for a Bleeding Fibro-Myoma of the Uterus 481
Jaggard, W. W., A Puerperal Uterus Showing Endometritis Puerperalis 479
Jaggard, W. W., Chronic Inversion of the Uterus 69
Jewell, Prof. J. S., Obituary 533
Johnson, F. S., Medical Matters in Havana, Cuba —
Johnson, H. A., Pneumatic Differentiation and Medication, 26-203
Chronic Pseudo-Membranous Bronchitis 30-201

K.

Kidney, Digital Exploration of the 476
Knapp, H., Comparison of the Methods of Removal of Cataract with and without Iridectomy 525
Kuh, E. J., Asthma, Etiology and Cure of 279

L.

Lagorio, A., Foreign Correspondence —
Theory of Prof. Semnoa of Bright's Disease 55

PAGE

Lagorio, A., Foreign Correspondence
 Annual Meeting of the Italian Surgical Society 456
Larynx; Deep Tubing of the, as a Substitute for Intubation 407
Lupus Vulgaris 161

M.

Manual of Operative Surgery, Bryant 543
Martin, F. H., Electrolysis in the Treatment of Fibroid Tumors of the Uterus 203
Hystero-Neurasthenia 405
Mattison, J. B., Cocaine-Dosage and Cocaine Addiction 335
Matters of Interest 323
Meany, W. B., Notes from London, England 364
Medical Association, Annual Meeting of the American 323
Medical Congress Ninth International 95, 423
Medical Congress, Ninth International, Addresses in 535
Medical Electricity: Its Application to Medicine and Surgery 631
Medical Matters in Havana, Cuba 367
Medical Notes 537
Medical Societies to Meet in Chicago 536
Medicine in Paris and Vienna 245
Meningitis, A Pathognomonic Symptom in Tubercular 41
 A Case of Miliary Tubercular 404
Metritis 97
Mortality in the Medical Profession 144

N.

Nasal Duct, Stricture, Modifications of the Treatment of 291
Nephritis, Chronic Diffuse 15
Notes from a London Clinic 553
Notes from London, England 364
The Neurological Review 211

O.

Obstetrics. A Case of 263
Obstetrical Pads of Garrigues and Richardson, The Antiseptic 376

PAGE

Ocular and Nasal Diseases, Connection Between 290
Ophthalmia, Sympathetic 291
Orton, Harlow N., A Case of Perinephritic Abscess 370
Our Penal Machinery and its Victims, J. P. Altgeld 91
Outlines of the Pathology and Treatment of Syphilis and Allied Diseases 629
Ovarian Adenoma with Uterus, Suppurating 173
Ovarian Cyst in Broad Ligament Containing Degenerated Ovary 172
Ovarian Cyst, Unicular of Right Ovary 175
Ovarian Cystoma Proliferating 171
Ovary, Dermoid Cysts of 377

P.

Pancreas, The Surgery of 425
Perforations of the Membrana Tympani, on the Management of 527
Park, A. V., a Case of Antepartum Hæmorrhage at Term 286
 A Case of Pyelitis of Nineteen Years' Standing, caused by Renal Calculus: Recovery 287
Parovarian Cyst and Appendages, Complicated by a Uterine Fibroid 172
Patella Tenden Reflex, Significance of 47
 Absence of 61
Patton, W. R., Pseudo-Membranous Bronchitis 139
Pelvic Inflammations, Antiseptic Tamponnement in the Treatment of 277
Pharynx, Diseases of the Vault of 18
Photography and Medicine 550
Placenta, Showing Veamatous Insertion of the Umbilical Cord, etc 175
Placenta, with Marginal Insertion of Short Umbilical Cord 376
Pneumatic Differentiation and Medication 26, 203
Practical Medicine, A System of, W. Pepper and L. Starr 92
Prostatic Myoma, Removed by Supra-Pubic Prostatomy, a Case of 403

PAGE

Pregnancy, Intertstitial, A Case of, with Removal, etc ... 259
Publishers' Drawer ... 449
Purdy, C. W., Stropanthus Hispidus : Its Pharmacology and Therapeutics ... 213
Pyelitis, a Case of Nineteen Years' Standing, caused by Renal Calculus : Recovery ... 287

R.

Rectal Medication by Means of Gases ... 362
Recurrent Hemorrhage into the Anterior Chamber ... 169
Report (Annual) of the Museum of Hygiene of the United States Navy ... 305
Reports and Pamphlets Received ... 94, 333, 447, 635
Retroperitoneal Cysts, The Operative Treatment of, Together with Mikulicz' System of Drainage ... 481
Reynaud's Disease, A Case of ... 1
Robison, J. A., Antipyrin and Thallin ... 10, 195
Climatic Treatment of Disease ... 299
Rush, Dr. Benjamin, The Monument to ... 535

S.

Salpingitis, Aetiology, Pathology and Classification of ... 181
Skeer, J. D., a Pathogonomonic Symptom in Tubercular Meningitis ... 41
Societies, Medical, to meet in Chicago ... 536
Spaying in Nervous Disease ... 49
Starkey, H. M., Modifications of the Treatment of Stricture of the Nasal Duct ... 291
Steele, D. A. K., Intus-susception in Infants ... 469
Stricture of the Nasal Duct, Modifications of the Treatment of ... 291
Strophanthus (Hispidus), Its Pharmacology and Therapeutics ... 23
Strophanthus-Lanoline ... 538
Stropanthus, Concerning the Cost of ... 537

PAGE

Surgery on the Continent of Europe ... 148
Surgical Diseases of the Kidney, Morris ... 330

T.

Teeth, Artificial, a Case of Swallowing a Set of ... 539
Tendon Reflex, Significance of Patella ... 47
Tents, Slippery Elm ... 373
The Illinois State Board of Health ... 145
The Poisonous Action of Potassium Chlorate ... 569
The Science and Art of Obstetrics, Parvin ... 326
The Surgical Diseases of Children, Owen ... 329
The Water Treatment in Typhoid Fever ... 455
Thirteenth Annual Report of the Secretary of the State Board of Health of Michigan 444
Transactions of the American Medical Association ... 438
Transactions of the Association of American Physicians ... 631
Transactions of the State Medical Society of Wisconsin ... 543
Transactions of the Texas Medical Association Eighteenth Annual Session ... 543
Transactions of the Thirty-sixth Session of the Illinois Medical Society ... 544
Tucker, J. I., Case of Herpes Omo-Brachialis. ... 237
Hydrophobia Vera ... 545
Tumor, A Case of Intra-Ocular 290
Turner, Thomas J., Annual Report of the Museum of Hygiene of the United States Navy ... 305
Typhoid Fever, Concerning the Present Standing of the Water Treatment of ... 468
Typhoid Fever, Ilium of Child Showing Characteristic Lesions ... 176
Thymol in the Treatment of Atropic Nasal Catarrh ... 562
Tympani, Membrana, See Perforations.

U.

PAGE

Uterus, Chronic Inversion of 69
Puerperal, Showing Endometritis Puerperalis 479
Uterus, Electrolysis in the Treatment of Fibroid Tumors of 203
Uterus, Lungs and other Organs, Fibro-Sarcoma of 174
Uterus, Removal of the Ovaries and Tubes for Bleeding, Myoma of the 481

V.

Vagina, Antiseptic Tamponnement in the Treatment of Pelvic Inflammations 277
Vaginal Enterocele, Anterior 178
Vaginal Pressure in Treatment of Chronic Pelvic Disease —

W.

Ware, Lyman, Lupus Vulgaris, 161
A Ciinical Study of Glaucoma 288
Warming-Crib, The 449
Waxham, F. E., Modified Intubation Instruments 300
Wear and Tear, or Hints for the Overworked, Mitchell 544
Wenzel, H. P., Thoracic Aneurism 111
Weston, E. B., Metritis 97
Wing, Elbert, Exhibition of Heart with Atheroma 285
Exhibition of Specimen Illustrating Differential Diagnosis between Cavities in Tuberculosis and Bronchiectosis 286
Wing, Elbert, Two Recent Autopsies at Cook County Hospital 416
Wing, Elbert, Concerning the Present Standing of the Water Treatment of Typhoid Fever, Abstract —

Z.

Zeisler, J., Ichthyol in the Treatment of Skin Diseases 66

### PAMPHLETS RECEIVED.

*A Successful Case of Partial Excision of the Larynx on account of Intra-Larnyngeal Epithelioma.* By Lennox Browne, F. R. C. S. [Ed.]

*A Discussion of the Climatic Treatment of Phthisis Pulmonalis.* By Joseph P. Ross, A. M., M. D.

*Novel Method of Treating Diseases of the Middle Ear.* By Seth H. Bishop., M. D.

*Surgical Lesions of the Brain and its Envelopes.* By Nicholas Senn M. D.

*Hydrophobia—Fatal Termination.* By H. P. Stebbings, M. D.

*A Contribution to the Management of General Atrophy (Sclerosis?) of the Conducting Apparatus of the Ear.* By S. O. Richey, M. D.

*Persistent Pain after Abdominal Section.* By James B. Hunter, M. D.

*The Scientific Rationale of Electro-therapy.* By C. H. Hughes, M. D.

*Post-Graduate Instruction in Gynæcology.* By Henry C. Coe, M. D., M. R. C. S.

*Ueber das Vorkommen der Albuminuria bei Diabetes Mellitus.* Von Dr. Arnold Pollatschek.

*A Novel System of Operating for the Correction of the Deflected Septum.* By William C. Jarvis, M. D.

*Forensic Surgery.* William Zuppan versus William Dickinson, M. D.

*Baked Beans; A Serio-Humorous Medical Paper.* By Ephraim Cutler, A. M., M. D.

*Will Contests.* By Walter E. Rex, Esq.

*Live-Birth in Its Medico-Legal Relations.* By John J. Reese, M. D.

### BOOKS RECEIVED.

*Report of the Committee on Disinfectants of the American Public Health Association.* 1886.

*Proceedings and Addresses at a Sanitary Convention held at Coldwater, Michigan, Sept. 9th and 10th, 1886.*

*A Compend of Electricity and Its Medical and Surgical Uses.* By Charles F. Mason, M.D.

*A Treatise on Diseases of the Skin.* By T. M'Call Anderson, M.D.

*A Manual of Weights and Measures, Etc.* By Oscar Oldberg, Pharm. D.

*The Pathology of Pregnancy.* Vol. II. of a *Practical Treatise on Obstetrics*, By A. Charpentier, M.D.; and *The Pathology of Labor*, Vol. III. of same Treatise. Both volumes number in the *Cyclopædia of Obstetrics and Gynæcology.*

*The Nursing and Care of the Nervous and Insane.* By Charles K. Mills, M.D.

*Drug Eruptions, a Clinical Study, Etc.* By Prince A. Morrow, A.M., M.D.

*Reference Handbook of the Medical Sciences.* Vol. IV.

*A Text-book of Pathological Anatomy and Pathogenesis.* By Prof. Ernst Ziegler.

*A Companion to the United States Pharmacopœia, Second Revised Edition.* By Oscar Oldberg, Pharm. D., and Otto A. Wall, M.D., Ph. G.

*Earth as a Topical Application in Surgery.* By Addinell Hewson, M.D.

*Elements of Physiological Psychology.* By Prof. George T. Ladd (Yale).

*The Practitioner's Handbook of Treatment.* By J. Milner Fothergill, M.D.

*Medical Electricity, Etc.; Third Edition, etc.* By Robert Bartholow, A.M., M.D., LL.D.

*A Practical Treatise on Impotence, Sterility, and Allied Disorders of the Male Sexual Organs.* By Samuel W. Gross, A.M., M.D., LL.D.

*Saint Bartholomew's Hospital Reports.* Vol. XXII.

*Report of the Commissioners of Education for the Year 1884–5.*

Index etc

SUBSCRIPTION PRICE, $3,00 PER YEAR, IN ADVANCE.

Whole Number, 330 JUNE, 1887. Vol. LIV., Number 6.

LIBRARY INDEXED S.G.O.

### THE CHICAGO MEDICAL JOURNAL AND EXAMINER

Editor: S. J. JONES, M.D., LL.D.

COLLABORATORS.

CHICAGO.

PROFESSOR E. ANDREWS, M.D., LL.D.
PROFESSOR J. BARTLETT, M.D.
PROFESSOR W. H. BYFORD, A.M., M.D.
PROFESSOR L. CURTIS, A.M., M.D.
PROFESSOR O. C. DEWOLF, A.M., M.D.
PROFESSOR E. C. DUDLEY, A.B., M.D.
PROFESSOR C. FENGER, M.D.
PROFESSOR ELBERT WING, A.M., M.D.
PROFESSOR J. N. HYDE, A.M., M.D.
PROFESSOR E. F. INGALS, A.M., M.D.
PROFESSOR W. W. JAGGARD, A.M., M.D.
PROFESSOR F. S. JOHNSON, A.M., M.D.
PROFESSOR H. A. JOHNSON, M.D., LL.D.
PROFESSOR C. W. PURDY, M.D.
PROFESSOR C. G. SMITH, A.M., M.D.
PROFESSOR F. BILLINGS, M.D.
PROFESSOR W. T. BELFIELD, M.D.

MILWAUKEE, WISCONSIN:
PROFESSOR N. SENN, M.D.

WASHINGTON, D. C.:
S. O. RICHEY, M.D.

UNITED STATES ARMY:
SURGEON D. L. HUNTINGTON, M.D.

UNITED STATES NAVY
MEDICAL DIRECTOR J. M. BROWNE, M.D.
MEDICAL DIRECTOR A. L. GIHON, A.M., M.D.

